# Accuracy of Outcome Ascertainment in Long-Term Mortality After Coronary Artery Bypass Grafting

**DOI:** 10.1016/j.mayocpiqo.2024.08.006

**Published:** 2024-10-08

**Authors:** Kensuke Yokoi, Atsushi Tanaka, Goro Yoshioka, Masahiko Hara, Keiji Kamohara, Koichi Node

**Affiliations:** a Department of Cardiovascular Medicine, Saga University, Saga, Japan; b Department of Clinical Investigation, Japan Society of Clinical Research, Osaka, Japan; c Department of Thoracic and Cardiovascular Surgery, Saga University, Saga, Japan

## Abstract

Long-term outcome ascertainment can be affected by the follow-up performance and needs to use a different data source for more comprehensive data capture. However, a universal tracking system is absent in Japan, and long-term outcomes are often ascertained through electronic medical records (EMRs), the reliability of which is uncertain. In this study, we compared EMR-based and direct outreach–based collections on outcome ascertainment accuracy in 500 patients who underwent coronary artery bypass grafting. Mortality data for all patients were extracted from the EMR, as standard data collection. When patient death was not confirmed in the EMR, we enhanced to collect updated mortality information by direct outreach to patients, their family, or their physicians, as enhanced direct outreach data. As a result, the Kaplan-Meier curves found a notable separation between different data sources analyzed. Interestingly, mortality events in the latter half of the follow-up period (median, 6.5 years) were overestimated in the EMR-based data collection analysis because of the reduced number of actively tracked cases, highlighting a potential bias in the EMR-based data collection on long-term prognoses. Our findings suggest that an active follow-up strategy with better adherence will enhance the accuracy of long-term outcome ascertainment and be helpful to build more reliable real-world evidence.

Clinical trials in patients with, but not limited to, coronary artery disease focus on long follow-up periods to obtain comprehensive data in clinical decision-making.^1^^,^^2^ However, long durations in randomized clinical trial largely increase the required cost and effort, and the primary end points for both the Synergy between PCI with Taxus and Cardiac Surgery and the International Study of Comparative Health Effectiveness with Medical and Invasive Approaches trials were not as long as those reported in the long-term observational studies.^3^^,^^4^ Real-world evidence, which is generated through real-world data (RWD) analysis, potentially serves to complement the interpretation of results obtained from randomized clinical trials over extended periods in the real-world settings.^5^

The RWD are often collected from electronic medical records (EMRs); therefore, the comprehensiveness of follow-up in EMR is important in the utility of RWD to better understand clinical outcomes. However, this long-term outcome information can be affected by the follow-up extent and adherence. The EMR-based RWD may be insufficient for long-term event ascertainment, and long-term observational studies need to use a different data source for more comprehensive data capture, as that performed by using claim data in the United States. However, a universal system is absent in Japan, and long-term outcomes are often ascertained through EMR, the reliability of which is uncertain. Therefore, we compared direct outreach–based and EMR-based collections on outcome ascertainment change in evaluating long-term mortality data in patients who underwent coronary artery bypass grafting (CABG).

## Patients and Methods

This retrospective study analyzed 500 consecutive patients who underwent successful CABG at Saga University Hospital in Japan between February 2008 and December 2020. Mortality data for all patients were extracted from the EMR at Saga University, as standard data collection. When patient death was not confirmed in the EMR, we enhanced to collect updated mortality information by direct outreach to patients, their family, or their physicians, as enhanced direct outreach (EDO) data. The follow-up duration and number of deaths in the entire cohort of 500 patients were examined and compared between the EMR-based and EDO-based prognostic data. Kaplan-Meier method was used to estimate the mortality rate, and differences between groups were compared using log-rank test. All statistical analyses were performed using R software (version 4.3.2). Ethical approval was obtained from the Ethics Committee of the Saga University Hospital (20220201). The requirement for informed consent from individual participants was waived owing to the retrospective study design and minimal risk.

## Results

At baseline, the mean age was 68.1±10.1 years, and 76.2% of participants were male; 23.6% were on angiotensin-converting enzyme inhibitors/angiotensin-receptor blockers, 71.4% on β-blockers, and 49.2% on statins. Mean left ventricular ejection fraction was 59.1%±14.4%. In the EMR-based analysis of 500 patients who underwent CABG, 130 deaths were recorded over a median follow-up period of 3.9 years (interquartile range, 1.1-6.5 years). However, for the 370 patients whose deaths were not confirmed in the EMR, the EDO-based collection allowed for their extended follow-up. This approach increased the median follow-up duration to 6.5 years (interquartile range, 3.2-9.6 years) and revealed an additional 60 deaths, raising the total to 190. Event curves were almost consistent up to the initial median follow-up of 3.9 years as per the EMR-based data (Figure A), beyond which the curves gradually diverged with the EDO-based data, showing a downward trend in cumulative mortality. Excluded cases with a follow-up period of less than 3.9 years (the median follow-up in the EMR-based data), the landmark analysis beginning at 3.9 years found statistically significant differences between the 2 data sources (*P*=.03) (Figure B).

**Figure:**
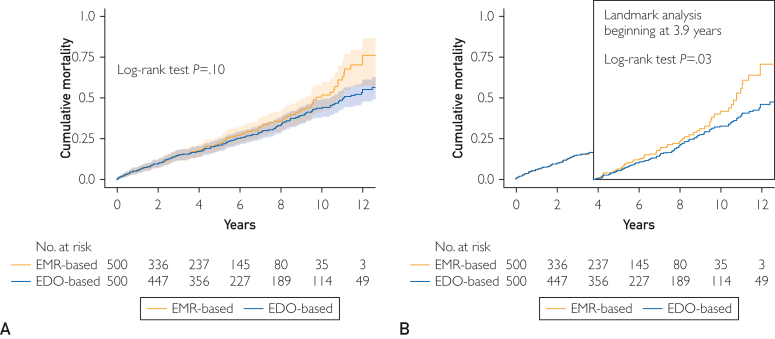
Comparative analysis of cumulative mortality between EMR and EDO data collection. A, Overall study duration. B, Landmark analysis after the initial median follow-up period (3.9 years). EDO, enhanced direct outreach; EMR, electronic medical record.

## Discussion

To our knowledge, this is the first study to compare the long-term mortality data collected by different methods in patients who underwent CABG. In general, the later phase of the survival curves often shows substantial slope changes with a wide CI, and this may be due to reduced number of tracked cases. This brought us a research question about the reliability of the survival curves at the later phase of follow-up period. To address this issue, in this study, we generated another cohort with an increased tracking rate using EDO method and compared its mortality rate with an original EMR-based data. However, because the tracking rate at the earlier phase of follow-up period was relatively kept in both data sets, we further conducted a landmark analysis beginning at 3.9 years (the median follow-up in the EMR-based data) to assess the reliability at the later phase of EMR-based mortality data. Despite analyzing the same cohort, the survival curves found a notable separation between different data sources analyzed. Thus, mortality rate in the latter half of the follow-up period was overestimated when the EMR-based collection was used because of the reduced number of actively tracked cases. This resulted in an upward adjustment in the cumulative mortality estimates from the original event curve, highlighting a potential bias in the EMR-based data collection on long-term prognoses. Our findings suggest that careful attention to the follow-up method and its adherence, as well as tracking rates by assessing the number at risk, when interpreting the mortality rates especially in the RWD is needed.

A key limitation of this study is that it was a single-center confirmatory study with a limited sample size, and further large-scale research is therefore required to validate our findings in other institutions and regions. Although a direct outreach was implemented in this study to assess whether the patients who were lost to follow-up were alive, a reliable and universal data-sharing platform may be potent to improve the active-tracking rates. Further research is also required to explore a better approach to enhance the accuracy of data collection on nonmortality outcomes because the reliability of nonmortality outcomes based on report from family members or physicians would be expected to be lower than that of death.

## Conclusion

In conclusion, an enhanced follow-up strategy with better adherence is needed to enhance the accuracy of long-term outcome ascertainment.

## Potential Competing Interests

The authors report no competing interests.

## Ethics Statement

Ethical approval was obtained from the Ethics Committee of the Saga University Hospital (20220201). The requirement for informed consent from individual participants was waived owing to the retrospective study design and minimal risk.
